# The beginning of a beautiful friendship: Cross-linking/mass spectrometry and modelling of proteins and multi-protein complexes

**DOI:** 10.1016/j.jsb.2010.10.014

**Published:** 2011-03

**Authors:** Juri Rappsilber

**Affiliations:** Wellcome Trust Centre for Cell Biology, University of Edinburgh, Michael Swann Building, King’s Buildings, Mayfield Road, Edinburgh, EH9 3JR Scotland, UK

**Keywords:** Cross-linking, Mass spectrometry, Modelling, Integrated structural biology, Multi-protein complexes

## Abstract

After more than a decade of method development, cross-linking in combination with mass spectrometry and bioinformatics is finally coming of age. This technology now provides improved opportunities for modelling by mapping structural details of functional complexes in solution. The structure of proteins or protein complexes is ascertained by identifying amino acid pairs that are positioned in close proximity to each other. The validity of this technique has recently been benchmarked for large multi-protein complexes, by comparing cross-link data with that from a crystal structure of RNA polymerase II. Here, the specific nature of this cross-linking data will be discussed to assess the technical challenges and opportunities for model building. We believe that once remaining technological challenges of cross-linking/mass spectrometry have been addressed and cross-linking/mass spectrometry data has been incorporated into modelling algorithms it will quickly become an indispensable companion of protein and protein complex modelling and a corner-stone of integrated structural biology.

## 1 Introduction

### 1.1 Background

Cross-linking converts non-covalent interactions between proteins or simply their proximity into covalent bonds. The artificially fused molecules withstand denaturating conditions and thus can be analysed using methods that normally dissociate protein complexes. As early as in the 1970s, this revealed protein–protein contacts in ribosomes through the pairing of cross-linking with gel electrophoretic approaches (Clegg and Hayes, 1974; Sun et al., 1974). Nearly 30 years later, the arrival of peptide mass spectrometry (MS) and its transforming powers on all fields of life sciences (Aebersold and Mann, 2003) provided the impetus to develop cross-linking methods (reviewed by (Back et al., 2003; Trakselis et al., 2005; Sinz, 2006, 2007; Mouradov et al., 2008; Tang and Bruce, 2009; Singh et al., 2010)). MS promised to efficiently identify the cross-linked proteins and furthermore to reveal precisely which residues were involved in the cross-link. In order to be cross-linked, residues must be within a certain distance of each other, (determined by the cross-linking agent used), and a pair of residues being cross-linkable therefore provides a valuable experimental constraint for any modelling attempt.

Cross-linking and MS were used to provide a topological map of the Nup84 complex by gel electrophoretically separating and identifying cross-linked proteins (Rappsilber et al., 2000). This approach has since been confirmed by the crystallographically characterized yeast 20S proteasome core (Denison and Kodadek, 2004) and been used for the analysis of the 19S proteasome lid (Sharon et al., 2006). These studies have shown the method to be a fast and reliable tool of proteomics, relying on protein identification as an established technology. A similar level of protein pair-wise interactions can also be obtained in a complementary way analysing native complexes by MS (Benesch and Robinson, 2006; Benesch et al., 2007; Heck, 2008).

Knowledge of the actual linkage sites, however, would increase the resolution of the method for structure determination from proteins to domains or even smaller sections, recently dubbed “peptide-level resolution” (Chen et al., 2010). Accordingly, cross-linked amino acids were identified and used as distance constraints in conjunction with threading to determine the fold of a protein or protein domain (Young et al., 2000). Mass spectrometers, protocols, and algorithms have advanced since these first experiments a decade ago, such that cross-linking/MS can now be employed for the structural analysis of multi-protein complexes, even if a complex proved challenging by other methods. Complexes that have been studied using these tools range from protein-peptide to large multi-protein complexes and include the Ffh.FtsY complex (Chu et al., 2004), GRP94 (Chu et al., 2006b), the Ndc80 complex (Maiolica et al., 2007), the annexin A2/p11 complex (Schulz et al., 2007), an epitope–antibody complex (Pimenova et al., 2008), the calmodulin–Munc13 complex (Dimova et al., 2009), the phi29 connector/scaffolding complex (Fu et al., 2010), the GroEL–GroES chaperonin complex (Trnka and Burlingame, 2010), and RNA polymerase II alone and in complex with transcription factor IIF (Chen et al., 2010) (discussed in Section 3).

### 1.2 Current experience with cross-linking/mass spectrometry

Cross-linking/MS has a number of strengths (for challenges see Section 4). First and foremost, the analysis takes place in solution and focuses on large structures, i.e. provides data on proteins and domains in their native and quaternary structure. Heterogeneity in the sample as a result of multiple conformations, complex populations with differing subunit composition, or presence of other proteins may lengthen the analysis time and challenge the data interpretation but does not principally impair the study. In pioneering studies, proteins have been cross-linked in bacterial whole cell lysates (Rinner et al., 2008) and cell membranes of living bacteria (Zhang et al., 2009). The method is applicable to a wide selection of structural motifs including the otherwise difficult to study coiled coils (Maiolica et al., 2007) and possibly partially unfolded regions, although some folding appears to be required for cross-linking to take place (Chen et al., 2010). Also, conformational changes in proteins have been studied in solution as compared to the crystal structure for the membrane protein rhodopsin (Jacobsen et al., 2006) or induced by binding of small molecules (Muller et al., 2009). Finally, cross-linking is fast and economical, and mass spectrometers are widely available for proteomic applications. Developers around the globe are tackling the current bottleneck of cross-linking/MS, namely the computational search tools for the identification of cross-linked peptides (see Section 2.4).

### 1.3 A potential link between cross-linking/mass spectrometry and modelling

Cross-linking/MS data have been used in conjunction with modelling, for example, to support homology modelling (Young et al., 2000; Chen et al., 2010) and to expand the crystal structure of the stable Pol II core towards the more dynamic periphery of a bound transcription factor, TFIIF (Chen et al., 2010). These and other individual applications (Fu et al., 2010) lack an automated framework. Nevertheless improvements in cross-linking/MS methods are expected to expedite many if not all aspects of modelling. Platforms to link cross-link data and modelling are being developed (Heymann et al., 2008). Model building based on X-ray diffraction may benefit when fitting protein chains into patchy regions of a density map or positioning un-observed protein regions such as loops, trimmed or truncated sequences, or missing sub-units. Docking of proteins may move from binary systems using e.g. HADDOCK (Karaca et al., 2010) to larger systems. Similarly, cross-link data may provide the intermediate resolution range currently lacking for the reconstruction of multi-domain proteins or multi-protein complexes from individual structure fragments obtained by X-ray crystallography, nuclear magnetic resonance (NMR), or homology modelling and out-line/shape revealing methods such as electron microscopic approaches or small angle X-ray scattering. In even larger assemblies, cross-linking may substitute for protein co-purification data such as used in reconstructing the nuclear pore complex (Alber et al., 2007a,b). Last but not least, cross-linking opens a road towards dynamic aspects of proteins and multi-protein complexes. As an example, conformational changes could be modelled starting from a high-resolution structure of one conformation and a cross-linking/MS analysis of another conformation. This list will hopefully motivate developers of modelling tools to integrate cross-linking/MS data into the modelling process to reduce this current “bottleneck”.

Progression from early proof-of-concept experiments to the advent of routine application of cross-linking in structural biology requires a number of key challenges to be addressed. The experimental workflow of cross-linking/mass spectrometry will be outlined here. Discussion of the results of a recent detailed analysis of two large multi-protein complexes, Pol II and Pol II-TFIIF, will highlight practical details of the cross-link approach. Finally for researchers planning an experiment, interpreting results or using data for modelling a set of conclusions will be presented that summarize current knowledge on cross-link data.

## 2 Analytical workflow of cross-linking/mass spectrometry

**Fig.1:**
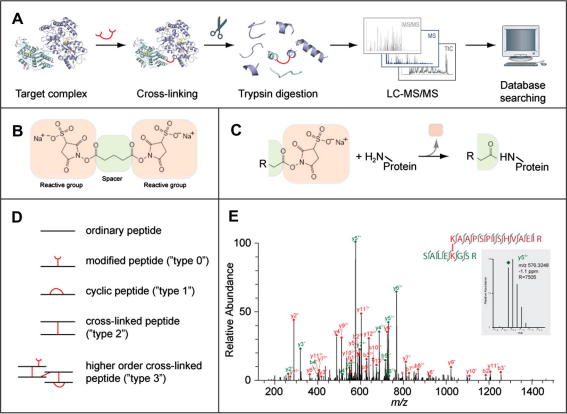
(A) Outline of the cross-linking/mass spectrometry process. A target complex is cross-linked in solution and digested with trypsin into peptides. The peptides are analysed by liquid chromatography coupled high-resolution mass spectrometry (LC–MS/MS) to obtain high-resolution masses and fragment masses (high/high) for cross-linked peptides. The fragmentation spectra of all peptides are subjected to database searching to identify cross-linked peptides. As an optional step, cross-linked peptides can be enriched before their LC–MS analysis. (B) A typical cross-linker, here bis(sulfosuccinimidyl)glutarate (BS2G), is composed of two reactive groups on either end separated by a spacer. This cross-linker reacts with primary amines (lysine side chain, protein N-terminus). Others target thiols (cysteine side chain) or activate carboxylic acids (aspartate, glutamate, protein C-terminus) for reaction with primary amines. (C) Reaction of a cross-linker with a primary amine. Part of the cross-linker, the leaving group, is replaced by the primary amine to form a covalent bond between the spacer and the amine. In this case, a peptide bond is formed. R can stand for either the rest of the cross-linker or may contain another protein, if the cross-linker had already reacted on its other end. (D) Peptides types that can be observed after cross-linking and trypsin digestion. (E) High resolution fragmentation spectrum of a cross-linked peptide obtained on an LTQ-Orbitrap mass spectrometer (adapted from (Chen et al., 2010)). Fragment peaks are annotated in red or green, depending on the peptide that fragmented and following the naming convention for peptides (y, C-terminal fragment; b, N-terminal fragment; both as a result of dissociating the peptide bond in the peptide back bone, followed by the number of amino acids included in the fragment and the charge of the fragment). All observed fragments are also indicated as bond cleavages between amino acids in the two cross-linked peptides. In this case, virtually all possible fragments of the peptide pair have been matched and virtually all peaks have been annotated resulting in a high-confidence identification of this cross-link. The inset shows a zoom onto one fragment peak (*m/z* 576, 3248) which matched with −1.1 ppm to the proposed peptide sequence. The high resolution of the spectrum (R 7505 for this peak) allows clear separation of the isotope peaks and consequently assignment of the fragment’s charge state.

The basic workflow to yield structural information of proteins and protein complexes by cross-linking/mass spectrometry (MS) is composed of four steps (Fig. 1A). Proteins are cross-linked in solution and then digested by trypsin to give peptides, some of which will be cross-linked. This mixture of peptides is then analysed by mass spectrometry and resulting data is interpreted to identify cross-linked peptides and determine the linked residues.

### 2.1 Protein cross-linking

Proteins are typically cross-linked in a chemical reaction involving a cross-linker and side chains of amino acids. The reactivity of amino groups, thiols and carboxylic acids render them as prime targets for cross-linking. The cross-linker is typically a molecule with two reactive groups on either end, separated by a spacer (Fig. 1B). These reactive groups can target either primary amino groups (found in the side chain of lysine and at the protein N-terminus) (Fig. 1C) or thiols (cysteine side chain). In published work to date, cross-linkers exclusively targeting amino groups have been used in cross-linking/MS studies of multi-protein complexes due to the high frequency of lysine in proteins and the consequently increased chance of obtaining and identifying cross-links. Alternatively, photo-activatable groups can be used in a cross-linker with currently poorly defined but presumably lower specificity (Krauth et al., 2009; Gomes and Gozzo, 2010). The result is always that the cross-linker bridges between residues within a protein or between two proteins at a maximal distance influenced by the length of the spacer. In a single exception, a small molecule, 1-Ethyl-3-(3-dimethylaminopropyl)carbodiimide (EDC) is used to activate carboxylic acids (aspartate, glutamate, protein C-terminus) to cross-link with amines (lysine, protein N-terminus). This directly cross-links atoms of the protein(s) with each other in a “zero-length” cross-link. Cross-linkers with three reactive groups exist but have not yet been used in structural work as they greatly increase the analytical challenges involved in identifying the three cross-linked amino acid residues. Cross-linkers are commercially available from several companies. New cross-linkers are being developed with improved chemical (Bich et al., 2010) or mass spectrometric properties (Petrotchenko et al., 2005, 2009, 2010; Tang et al., 2005; Chowdhury et al., 2006; Ihling et al., 2006; Gardner et al., 2008; Lu et al., 2008; Krauth et al., 2009; Paramelle et al., 2009; Dreiocker et al., 2010; Liu and Goshe, 2010; Yang et al., 2010; Zelter et al., 2010).

### 2.2 Digestion of cross-linked proteins to peptides

The identification of cross-link sites employs the well-established workflows of proteomics, but with a twist. Proteins are digested by proteases, typically trypsin, into peptides which can be fractionated or separated but ultimately are analysed by mass spectrometry to determine their mass and usually also fragmentation spectra (Fig. 1A). Standard proteomics analysis deals only with linear peptides in its efforts to identify and quantify proteins and to determine their modification sites. To these, cross-linking adds a number of different species (Fig. 1D). At the protein level, cross-linking results in two products: a cross-link, when the cross-linker reacted with one amino acid on either end, or a modification, when the cross-linker reacted with an amino acid on one and water on the other end. At the peptide level, this can lead to three different situations and their combinations (Fig. 1D): modified peptides (type 0, nomenclature by (Schilling et al., 2003)), cyclic or internally bridged peptides (type 1), cross-linked peptides (type 2), or any combination of these (type 3). All of these peptides contain structural information. The current focus is on cross-linked peptides (type 2) as they contain long-distance information. In contrast, modified peptides (type 0) reflect accessibility while cyclic peptides (type 1) reveal information about local structure such as alpha-helical regions (Maiolica et al., 2007). Higher order cross-links (type 3) have yet to be observed and will likely be difficult to identify due to complex fragmentation spectra. Methods that distinguish during mass spectrometric detection between different cross-link products include isotope labelling schemes (Back et al., 2002; Chu et al., 2006a) and special cross-linker chemistry (Petrotchenko et al., 2005).

### 2.3 Mass spectrometric analysis of cross-linked peptides

MS provides the data to identify cross-linked residues in a two-staged process. First, the cross-linked peptide needs to be identified. For this, the mass and usually also the fragmentation spectrum of the cross-linked peptide have to be acquired and then analysed by database searching. Detailed analysis of the fragmentation spectrum may then reveal the exact or approximate sites of linkage, depending primarily on the quality and dynamic range of the spectrum. The analysis of peptide fragmentation spectra in general is simplified by the fact that peptides normally follow specific fragmentation rules, breaking predominantly along the backbone, at the peptide bond when using the most commonly employed fragmentation method, collision-induced dissociation (CID) (Wells and McLuckey, 2005). Peptide fragmentation by CID gives rise to two main fragment types, the N-terminal “b-ions” and the C-terminal “y-ions” (Roepstorff and Fohlman, 1984; Biemann, 1988). Peaks in fragmentation spectra are labelled using these letters, in conjunction with a subscript for the number of residues contained in the fragment and a superscript for the number and type (positive or negative) of charges of the ion. An alternative to CID is given by electron transfer dissociation (ETD) (Syka et al., 2004). In this case, c- and z-ions are observed predominantly, which origine from the cleavage of a different bond in the peptide backbone than the related b- and y-ions. Note that mass spectrometry measures the mass to charge ratio of ions. The charge of an ion can be determined from resolved isotope peaks and the mass then be calculated (for more details on peptide fragmentation in a mass spectrometer consult the introductory review written by Steen and Mann (2004)). Cross-linked peptides follow these general rules of peptide fragmentation by CID (Back et al., 2001; Schilling et al., 2003; Gaucher et al., 2006) and ETD (Chowdhury et al., 2009). The fragmentation spectrum of a cross-linked peptide typically features fragments of both cross-linked peptides (Fig. 1E) and can thus lead to the confident and unambiguous identification of both peptides. If a set of fragments is observed that fall upstream and downstream of the cross-linked residues, the exact position of the cross-linking site can be determined. In the spectrum displayed in Fig. 1E, the fragments that determine the linkage sites are red b1 and y13 and green y4/y5 and b4/b5, for the peptide sequences coloured correspondingly. Note that cross-linked peptides are best identified by high resolution measurement of peptide and fragment masses. This strategy, also called high–high, maximises the specificity of the database search. The identification of cross-linked peptides may furthermore be improved if specific reporter fragments are generated that are only observed in cross-linked peptides (Back et al., 2001; Seebacher et al., 2006; Iglesias et al., 2009, 2010) or new cross-linkers are used that guide the mass spectrometric analysis towards cross-linked peptides (Petrotchenko et al., 2005, 2009, 2010; Tang et al., 2005; Chowdhury et al., 2006; Ihling et al., 2006; Gardner et al., 2008; Lu et al., 2008; Krauth et al., 2009; Paramelle et al., 2009; Dreiocker et al., 2010; Liu and Goshe, 2010; Yang et al., 2010; Zelter et al., 2010).

For a long time, identifying cross-linked peptides was very challenging. The multiple possible cross-link products for any specific residue and typically incomplete cross-link reaction results in low signals for cross-linked peptides. These need to be detected against a high background of unmodified linear peptides and possibly also non-specific reaction by-products. Three elements worked together to recently address the data acquisition challenge of cross-linked peptides: enrichment of cross-linked peptides, improved mass spectrometers, and automated data interpretation.

Enriching cross-linked peptides improves their detection by MS and thus the yield in observed linkage sites. Various methods to achieve such an enrichment have been envisaged and are now being tested. One such approach that has so far been employed for the analysis of multi-protein complexes makes use of the generally higher charge state that distinguishes cross-linked peptides from linear peptides. This has been exploited, prior to acquisition, by cation-exchange chromatography which enriches cross-linked peptides carrying higher charges in the later eluting fractions (Rinner et al., 2008; Chen et al., 2010) and during acquisition on the MS where peptides with high charge states are selected for fragmentation (Maiolica et al., 2007; Rinner et al., 2008; Chen et al., 2010). Numerous other approaches are currently under development, particularly the use of cross-linkers that contain affinity groups for the selective enrichment of cross-linked peptides (Chu et al., 2006a; Kasper et al., 2007; Chowdhury et al., 2009; Kang et al., 2009; Nessen et al., 2009; Yan et al., 2009; Vellucci et al., 2010).

A new generation of mass spectrometers has increased the number of peptide species that can be selected for fragmentation in a single experiment, the sensitivity of their detection and the resolution of signals. This results in more of the low-intensity cross-linked peptides being included in the analysis and in high-quality fragmentation data that can be interpreted unambiguously. To deal with all this data efficiently, computational approaches have been developed that automate the data interpretation step and thus allow the power of liquid chromatography coupled mass spectrometry (LC–MS) to be used to create large data sets for the detection of cross-linked peptides. This is the subject of the next section.

### 2.4 Identification of cross-linked peptides

Cross-linked peptides can be identified using mass spectrometry analogously to linear peptides. For linear peptides, the peptide mass is taken to select candidate peptides from a protein database matching this mass within the experimental error. The fragmentation spectrum is then used to find, from among these candidates, the peptide sequence that best explains the observed fragment signals. To adopt the same workflow to cross-linked peptides, all possible cross-linked peptides must be predicted by in silico digestion of all proteins and then creation of all possible pair-wise combinations of peptides. Any peptide needs to be considered if it contains a residue that is capable of cross-linking in the actual experiment. The pairing leads to (*n*^2^ + *n*)/2 possible cross-links for *n* peptides. This *n*^2^ problem creates a challenge for search algorithms and the evaluation of any match between a spectrum and a candidate peptide pair due to the high risk of random matches in a large database. However, for protein complexes this problem is simplified, as only those proteins need to be considered that are actually present in the sample. The first automated algorithm that identified cross-links in a multi-protein complex (Maiolica et al., 2007) and the identification of cross-links in an *Escherichia coli* cell lysate (Rinner et al., 2008) revealed no limitations to database searching of cross-linked peptides in principle. A large number of algorithms and programmes to match spectra with candidate cross-linked peptides have been described (Schilling et al., 2003; de Koning et al., 2006; Gao et al., 2006; Maiolica et al., 2007; Heymann et al., 2008; Nadeau et al., 2008; Rinner et al., 2008; Singh et al., 2008; Chu et al., 2010; McIlwain et al., 2010; Petrotchenko and Borchers, 2010; Xu et al., 2010) and recently been reviewed (Leitner et al., 2010; Singh et al., 2010). Nevertheless, this is an area of ongoing developments not least because of a second challenge: determining the confidence of a match. False identifications of linear peptides have been reduced through manual interrogation of peptide-spectrum matches, by applying filters created using a training data set (Eng et al., 1994), using probabilistic approaches (Perkins et al., 1999; Nesvizhskii et al., 2003; Sadygov and Yates, 2003), relying on machine learning (Käll et al., 2007), and using the target-decoy approach, combining the ordinary (target) database usually with an inverted (decoy) database (Moore et al., 2002; Kislinger et al., 2003; Peng et al., 2003; Sennels et al., 2009). Following these experiences with linear peptides, the false positive rate of database searches for cross-linked peptides has been estimated by using the target-decoy method (Maiolica et al., 2007; Rinner et al., 2008), relying on a decoy database or using a false mass for the cross-linker, and manual interrogation following a decision tree (Chen et al., 2010).

## 3 RNA polymerase II complexes

### 3.1 RNA polymerase II core complex – benchmarking cross-linking/mass spectrometry

The analyses of protein complexes such as our success in using cross-linking/mass spectrometry to guide the engineering of a crystallisable *Homo sapiens* Ndc80^bonsai^ complex (Maiolica et al., 2007; Ciferri et al., 2008) demonstrated that the technology is in principle of value. However, a detailed analysis of the data’s accuracy was not possible, as all of these studies reported a relatively small number of linkage sites (10–25 at best). We therefore analysed recently (Chen et al., 2010) a large multi-protein complex, the *Saccharomyces cerevisiae* RNA polymerase II (Pol II), for which a crystal structure had been deposited (PDB 1WCM) (Armache et al., 2005) that could be used as a reference to check the quality of cross-link data.

**Fig.2:**
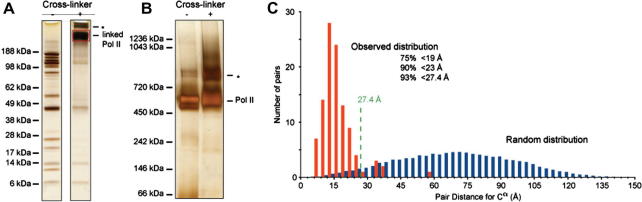
Benchmarking the cross-linking/mass spectrometry process using *S. cerevisiae* RNA polymerase II (Pol II) and its crystal structure. (A) The subunits of Pol II are separated by denaturating gel electrophoresis (SDS–PAGE) and visualized by silver staining. The individual subunits can be seen as separate bands before the addition of cross-linker (here bis(sulfosuccinimidyl)suberate (BS3)). After cross-linking, these individual bands disappear and a new, high-molecular weight band appears, corresponding to the cross-linked Pol II (red box). A higher molecular weight band corresponds possibly to Pol II dimers (asterisk). (B) Pol II migrates under native conditions mostly as a single band, both in the absence and presence of cross-linking. Under both conditions, some Pol II dimerization is observed (asterisk). (C) Distribution of alpha-carbon distances for lysine pairs in the crystal structure of Pol II (PDB 1WCM) (Armache et al., 2005) when scaling the distance distribution for all random lysine pairs in the crystal structure to 106 pairs (blue) and when taking the distance measure of those 106 pairs that were observed by cross-linking (red) (Chen et al., 2010). The predicted upper limit for cross-linkable lysine pairs in the crystal structure is here 27.4 Å. This upper limit includes the length of lysine side chains (2 × 6.5 Å), the length of the spacer (max. 11.4 Å) and an estimation of the positional error in the crystal structure (1.5 Å for surface residues). The upper limit does not consider the possibility of conformation changes or vibrations of the complex in solution. The observed distribution of cross-linked pairs is clearly not random and fulfils largely the theoretically predicted distance threshold for cross-linkable pairs. (All adapted from (Chen et al., 2010).)

Purified Pol II complexes (12 subunits, 513 kDa) cross-linked readily, as could be seen from the change in protein bands under denaturating gel electrophoresis before and after cross-linking (Fig. 2A) (Chen et al., 2010). We used the cross-linker bis(sulphosuccinimidyl)suberate (BS3, Thermo Fisher), which reacts with primary amines in lysine side chains and protein N-termini. Cross-linking did not lead to extensive aggregation of complexes, as could be seen from native gel electrophoresis (Fig. 2B). Thirty micrograms of Pol II were subjected to our analysis: cross-linking, gel electrophoresis to isolate monomeric complexes, trypsin digestion, fractionation of peptides by strong-cation exchange chromatography, liquid chromatography–mass spectrometry in a high–high strategy, and finally database searching to identify the cross-linked peptides. In summary, 429 fragmentation spectra matched to cross-linked peptides covering 146 unique linkage pairs. From this data, 106 linkage pairs were obtained for which distance data could be extracted from the crystal structure of Pol II. Following a decision tree the data supporting the 106 linkages was classified for its quality, leading to 80 higher-confidence and 26 lower-confidence cross-links.

The distance distribution for alpha-carbon pairs of cross-linked lysines was clearly different from a random set of lysine pairs selected from the crystal structure (Fig. 2C). Based on this comparison, two arguments could be made for the accuracy of the cross-link/mass spectrometry data. First, the observed distance distribution was very unlikely to be a random result (*P*-value of 3 × 10^−87^). Second, the observed distribution looked plausible when considering the length of a lysine side chain to be 6–6.5 Å, the length of the cross-linker in full extension to be 11.4 Å, and an experimental error for this crystal structure of 1–1.5 Å for surface residues (as estimated from the crystallographic B-factor). Adding all these together would predict the majority of cross-links to report lysine pairs whose alpha-carbons are closer than 27.4 Å in the crystal structure. This was indeed the case for 93% of the data.

With six of seven cross-links above 27 Å, the cross-links of longest length tended to fall into the mobile clamp domain of Pol II. These long distance cross-links could therefore be rationalised as capturing possible conformations of Pol II in solution. A single cross-link supposedly bridged residues whose alpha-carbons were positioned nearly 60 Å apart in the crystal structure. Dense packing of protein separates the residues according to the crystal structure, which makes conformational changes unlikely to bring these two residues into close enough proximity for cross-linking. Furthermore, the cross-link distance fell into the broad maximum of the randomly selected pairs and the data supporting the cross-link had been classified as being of lower confidence. We hence concluded that this single cross-link among the 106 observed cross-links was a false positive, suggesting a false positive rate of less than 1% when combining higher and lower confidence data.

### 3.2 RNA polymerase II-TFIIF – expanding a stable complex core towards its more elusive periphery

**Fig.3:**
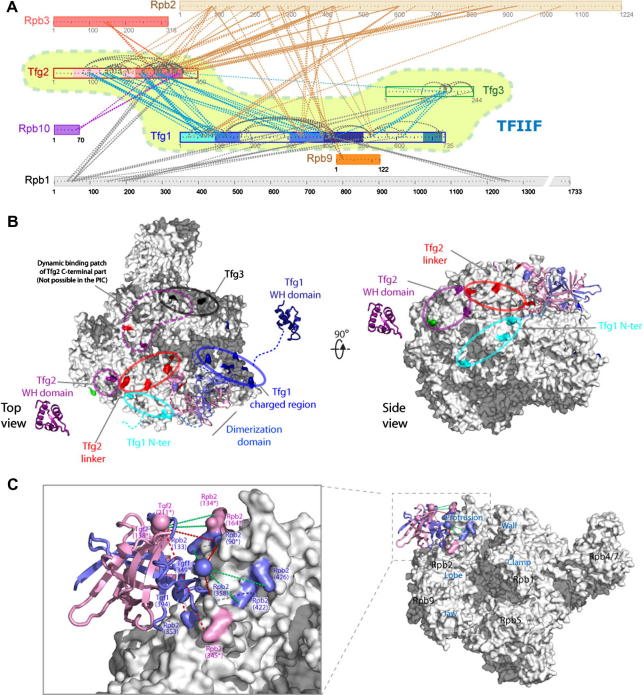
Cross-linking/mass spectrometry analysis of *S. cerevisiae* RNA polymerase II (Pol II) bound to transcription factor IIF (TFIIF). (A) Linkage map showing the sequence position of all observed cross-linked residue pairs within TFIIF and between TFIIF and Pol II. Connections between residues are blue within TFIIF or colour coded by Pol II subunit for cross-links between Pol II and TFIIF. Sequence regions of TFIIF subunits are colour coded (Tfg1: N-terminal tail, 2 × dimerization domain, charged region, winged-helix (WH) domain; Tfg2: 2 × dimerization domain, linker, WH domain). (B) Residues of Pol II colour coded by region in TFIIF subunits they cross-link with. (C) Homology model of the Tfg1–Tfg2 dimerization domain positioned on the Pol II structure (PDB 1WCM) with cross-linked residues labelled by proteins and residue number. Dashed lines connect pairs of residues that were used for the positioning, either because they were observed to cross-link or because they are the closest residues in the structure (denoted by an asterisk behind their residue number). (All adapted from (Chen et al., 2010).)

We next analysed the complex of Pol II with transcription factor IIF (TFIIF), comprising 15 subunits with a total molecular weight of 670 kDa, including the three subunits of TFIIF: Tfg1, Tfg2, and Tfg3 (Chen et al., 2010). A crystal structure of the Pol II complex and crystal structures for three domains of human TFIIF subunits composed the structural pre-knowledge. Using 200 μg of purified complex, and following the same strategy as outlined above, we identified 402 linkage sites within TFIIF or between Pol II and TFIIF. Cross-links within Pol II were observed but not evaluated. Using a decision tree as above, 224 higher-confidence cross-links were selected and used for model building. The data was summarized in form of a linkage map (Fig. 3A). This linkage map of the Pol II–TFIIF complex supported the validity of homology models for three TFIIF domains, provided a reciprocal footprint of TFIIF on Pol II and of Pol II on TFIIF at peptide resolution, and led to the docking of a homology model for the Tfg1/Tfg2 dimerisation domain of TFIIF with the Pol II crystal structure.

Homology modelling can be used to infer the structure of a novel protein or domain if the structure of a related protein or domain has already been determined at high resolution. Structures for the human winged-helix domains of Tfg1 and Tfg2 (Groft et al., 1998; Kamada et al., 2001) as well as for the Tfg1/Tfg2 dimerisation domain (Gaiser et al., 2000) had been solved and could be used as templates for homology modelling. The sequence alignment for the dimerization domain from *H. sapiens* and *S. cerevisiae* was not unambiguous, however, leaving an element of uncertainty on aspects of the model. Cross-links cannot currently be used to assist homology modelling. However the homology models obtained can be challenged by the experimental data. Indeed, the cross-link data for TFIIF was incorporated into the finished homology models in order to test if the model satisfied the experimental constraints.

The cross-link data between TFIIF and Pol II revealed the interaction regions between TFIIF and Pol II and located TFIIF on the Pol II surface (Fig. 3B). Cross-link sites in Pol II were colour coded by TFIIF region to visualize the footprint of individual TFIIF regions on the surface of Pol II. The data revealed distinct areas on Pol II that interact with the three TFIIF subunits. The position of different Tfg1 and Tfg2 regions could be followed in detail. For Tfg1, the N-terminal tail, dimerization domain and charged region were positioned on Pol II. For Tfg2, the dimerization domain, linker, and winged-helix domain were positioned on Pol II. Tfg3 is located in a region on Pol II that is occupied by other transcription factors in crystal structures of Pol II. This may indicate the importance of studying as fully assembled complexes as possible, because Pol II–TFIIF is only a sub-complex of the pre-initiation complex. Similarly, the C-terminal region of Tfg2 including the winged-helix domain displayed a number of alternative binding positions, some of which are not possible in the pre-initiation complex.

The mutually exclusive binding positions for the Tfg2 C-terminal region as revealed by cross-linking/MS demonstrated an ability of this technology to capture dynamic situations in protein complexes that is at the same time exciting and challenging. The fact that dynamic situations can be revealed by cross-linking/MS is exciting. The challenge is in the fact that the data of all different states of a complex or protein are superimposed. Utilizing the Pol II crystal structure has permitted the disentanglement of the overlying cross-link data and this has revealed the dynamic aspects of TFIIF binding.

Interestingly, the Tfg1 winged-helix domain was not found to link to any region of the Pol II–TFIIF complex other than the domain itself. This may indicate absence of specific interactions but not absence of random interactions as this domain is being held close to the rest of the complex by a linker region. Random interactions not being sufficient to lead to observable cross-links may indicate that transient interactions need to be long enough to provide the time required for the cross-linking reaction to take place and that the interactions have to represent a significant fraction of the population. In other words, a lower threshold for the stability of structures exists for them to be captured by cross-linking. Indeed, a recent study found a protein complexes with *K*(*D*) ∼ 25 μM to cross-link specifically while another complex with *K*(*D*) 100–300 μM did not, indicating the limit for cross-linking to be somewhere in this affinity range (Madler et al., 2010b).

The interaction between the Tfg1/Tfg2 dimerisation domain and Pol II surpassed this threshold and was observed by formation of numerous cross-links. This allowed docking of the domain and Pol II (Fig. 3C). As for the Tfg2 winged-helix domain, positional ambiguity resulted from cross-link data that could not be satisfied by a single binding mode. Taken together, this analysis found TFIIF binding to Pol II in multiple modes that possibly exchange in a dynamic fashion. The lessons learned from these analyses of two Pol II complexes are presented below, with regards to integrating of cross-linking/MS data into the modelling process as well as planning structural studies that utilize this technology.

## 4 Challenges of modelling when using cross-link data

The concept of a distance constraint is not new to modelling. Distance constraints are provided in large quantities for small proteins or domains in NMR (Nilges et al., 1997). Distance constraints are also obtained in small quantities for larger proteins and multi-protein complexes by other biophysical techniques, typically after introducing specific probes, e.g. spin labels in EPR (for review of low resolution methods and modelling (Venselaar et al., 2010)). Cross-link derived constraints are different from NMR data in being sparse and long distance. Even at “zero length” the length of the cross-linked side chains add to over 10 Å between the alpha-carbons of the linked residues. However, cross-linking yields constraints more plentiful and easier than any low-resolution biophysical method. A proper treatment of low-resolution distance constraints is now indicated for modelling. The following points at least should be considered when integrating cross-link data into modelling software.

### 4.1 Experimental data can be ambiguous

As an experimental method, cross-linking/MS will yield data with an experimental error. In our benchmarking experiment using the Pol II we found an experimental error of less than 1% with respect to miss-assigned linkages when combining high and low confidence data. None of the high confidence data proved to be incorrect, indicating that cross-linking/MS can yield unambiguous data. Nevertheless, there may also be value in lower confidence constraints, to reflect underrepresented conformers/structures or provide additional constraints for modelling. Thus, it would be desirable if modelling software could use constraint information while simultaneously taking their confidence level into account. In addition to this experimental error there is also positional ambiguity, when the site of linkage cannot be narrowed to a single residue but only a stretch of residues due to lack of fragmentation information. Alternatively, the same peptide sequence might be found in more than one location of a protein sequence or in more than one protein of a complex. This is particularly likely when detecting short peptides as partners in cross-linked peptides. Any modelling software should be able to deal with this ambiguity and reward models that satisfy at least one of the constraint alternatives.

### 4.2 From distance constraint to distance restraint

In first approximation, the distance constraint for the position of alpha-carbons in two cross-linked residues can be calculated by adding the length of the spacer in the cross-linker and the length of the linked side chains. This neglects, however, the dynamic behaviour of molecules in solution. Due to bond rotations and vibrations in the spacer, the cross-linker will sample a certain length distribution with the fully extended conformation being only one of many possible states. This has been modelled for a number of cross-linkers and a shorter “effective” length been proposed (Green et al., 2001). However, the protein(s) will also sample their conformational space in solution. The extent of residue movements will be protein and position dependent and as such is currently unpredictable. The influence of protein vibrations or conformational flexibility is likely to be of significantly larger importance than that of the cross-linker, especially when analysing large proteins or multi-protein complexes. Experimental data such as obtained for the Pol II may offer a heuristic solution to this problem. The amino acid pairs that were cross-linked did not spread equally over the range defined by the distance constraint. This suggests the possibility of using distance restraints instead of constraints. Using data obtained with cross-linkers of different length will improve the distance restraint by providing information on lower limits.

### 4.3 Cross-linking is undemocratic

Cross-linking/MS will result in more data for some parts of a protein or complex than in others. Several reasons account for this, and a number of approaches can be taken to obtain at least partial remedy of the undemocratic nature of cross-linking data. Firstly, cross-linking requires reactive sites in the protein(s) to be available, accessible, and in linkable geometry. Lysines have been consequently the target of choice, as they tend to be plentiful, accessible on the surface of proteins, and react with high specificity with *N*-hydroxysuccinimide cross-linkers (note that side reactions with serines, threonines and tyrosines have been observed (Leavell et al., 2004; Swaim et al., 2004; Kalkhof and Sinz, 2008; Madler et al., 2009, 2010a)). The distribution of lysine residues on the surface of proteins is, however, not even. Consequently, constraint data will vary in its coverage of a structure and be particularly scarce in hydrophobic regions such as hydrophobic cores or transmembrane regions. Experimentally, this can be addressed by targeting different residue pairs from lysine–lysine such as lysine–aspartate/glutamate, lysine–cysteine or cysteine–cysteine, all for which commercial cross-linking reagents are available. Also, use of photoactivatable linkers and even photoactivatable amino acid analogues such as azido-methionine or azido-leucine (Suchanek et al., 2005) or arginine–arginine cross-linkers (Zhang et al., 2008) are being explored. However, the patchy nature of cross-link data means that modelling will usually require additional structural data.

### 4.4 Absence of data is inconclusive but possibly suggestive

Not all cross-linked residues will actually be detected in cross-linked peptides. Contributing factors are the lower sensitivity of standard mass spectrometers in detecting larger peptides, masking of peaks by background and loss of hydrophobic peptides during the sample preparation. The apparent absence of an individual cross-link between two residues cannot justify the assumption that the two residues are not proximal. However, when considering groups of cross-links, absence of data might still be informative. For example, the presence of multiple cross-links between two regions A and B and the presence of multiple cross-links between two other regions C and D would indicate that cross-links in all four regions were observable in principle. Absence of cross-links between A/B and C/D would then indicate a lower probability of A/B and C/D to be proximal.

### 4.5 Population data

The protein(s) under investigation may be heterogeneous in a number of different ways. They will sample the conformational space available to them under the experimental conditions, i.e. not exist in one static structure. They may differ furthermore in their modification states, e.g. the presence and absence of a particular phosphorylation that may induce a large conformational change in a protein. On- and off-rates of subunits may lead to the presence of multiple forms of a complex. A particular strength of cross-linking is its ability to work with crude starting material. This means that even multiple forms of a complex may be present, such as assembly intermediates, fully assembled complexes but with different subunit composition to conduct specialised functions, or complexes at different processing states, differing in transient factors or conformations. Cross-linking will reflect this diversity to some extent and not just reflect a single static structure. No single model may therefore fulfil all constraints derived from cross-link data. Instead, modelling needs to create an ensemble of structures that relate to each other by conformational or compositional changes. Ultimately, this means that modelling will need to move closer to the structural reality by including dynamic aspects of proteins and protein complexes.

### 4.6 Artefacts

Cross-linking modifies proteins and in principle could result in structural artefacts in a number of ways, none of which has been shown to occur experimentally so far. An investigated protein could be caught in a very rare conformation, with further cross-linking events possibly exasperating the effect to create and therefore also reflect an otherwise impossible conformation. Similarly, a randomly passing protein might be caught to create a fusion that is not reflecting a functional protein–protein interaction. As cross-linking/MS provides data on populations these rare events have so far remained hidden among more frequent and less artefactual products of cross-linking. Cross-linking might furthermore trigger a conformation change that is either physiological but normally requires a stimulus such as binding of a co-factor or that is artefactual. At the current, possibly still initial state of comparing cross-linking/MS data with high-resolution structures, an extensive agreement between the two methods has been testified (Maiolica et al., 2007; Rinner et al., 2008; Chen et al., 2010). This argues at least against induced conformational changes being a frequent artefact of cross-linking. Finally, aggregates of proteins that arise from the high concentrations of protein solutions often used for cross-linking may be cross-linked and lead to artefactual protein–protein contacts. This can and has to be controlled for in each analysis by checking the analysed sample for cross-linked aggregates, for example using native gel electrophoresis (Chen et al., 2010). Besides these cross-linking artefacts there is also the possibility of erroneous data interpretation. This is possible, for example, during the database search (Section 2.4) or when considering ambiguous data during modelling (Section 4.1) and needs to be carefully controlled for. However, all currently available data indicate that cross-linking/MS is a reliable source of structural information.

### 4.7 Quantitative information

Cross-linking/mass spectrometry can provide quantitative data on the structure of proteins and complexes. It is tempting to speculate that this may pave the way for modelling to simulate dynamic structures. Quantitative information can in principle be obtained by comparing mass spectrometric signal intensities of different cross-links within an experiment and for identical cross-links in different experiments.

Comparing the yield for links across different experiments can be done by quantitative proteomics, relying on stable isotope labelling or label free approaches. Signal intensities for a cross-linked peptide or set of cross-linked peptides can be compared across different analyses. Alternatively, stable isotopes can be used to encode in a single analysis (typically leading to higher accuracy) the origin of peptides from different experiments, to allow using their relative peak intensities for quantitation. This approach is well established in proteomics and relies on incorporating stable isotopes, for example by amino acids in cell culture (SILAC) (Ong et al., 2002, 2003; Blagoev et al., 2003). Isotope-labelled chemical modifiers that react, for example, with the N-terminus of all peptides (iTRAQ and TMT) can be used alternatively. In addition, a pair of light and heavy labelled cross-linker might be used (Petrotchenko et al., 2005; Chu et al., 2006a; Ihling et al., 2006). For example, a protein could be cross-linked with a light cross-linker under one condition and with the heavy version of the same cross-linker under a different condition that may, for example, change the protein conformation. Mixing the two proteins and then analysing the cross-linked peptides by mass spectrometry will create signal pairs for every observable cross-linked peptide. The peak ratio of the pair gives the relative yield of this cross-link under the two conditions and thus reveals to what extent the cross-linked sites are affected by the condition change. Yield differences do not neccesarily directly relate to conformation changes but might also result from indirect effects such as changes in local competition for one site by proximal sites. For example, in conformation 1 site A links to site B and site C is distal; in conformation 2 site C becomes proximal to site A and competes with site B for the linkage. Therefore, whilst A–C’s change is clearly directly due to a conformational change, A–B’s change is a secondary effect albeit still being spatially near the primary effect.

Comparing the yield of different cross-links within an experiment is more challenging. Firstly, yield differences result from interplay of several environmental factors such as accessibility and reactivity of both sites, their relative position to each other in terms of orientation of side chains and possible obstructions, and conformational flexibility bringing sites into sufficient proximity possibly with only limited occurrence. In addition, peptides differ in their respective “flyability”, the intensity by which a given peptide is observed in a mass spectrometer. Different peptides can therefore not be compared individually with one another. This means that cross-linking does not provide any information on the abundance of an observed proximity. However, the “flyability” differences between peptides can be statistically averaged when comparing groups of peptides. This strategy is used when comparing the concentration of different proteins in the same sample (Rappsilber et al., 2002; Ishihama et al., 2005). Comparing, for example, groups of conformation-specific cross-links may well indicate the relative ratio by which the conformations occur.

## 5 Conclusion

After more than a decade of method developments and proof-of-concept studies, cross-linking/mass spectrometry is nearing the end of adolescence. The analysis of several multi-protein complexes shows this technology to yield rich constraint data that expedites the modelling process of proteins and multi-protein complexes. The technology itself may still need further consolidation, namely the development of a user-friendly and powerful database search engine. Also, more studies are needed to develop cross-linking/mass spectrometry into a generally accepted method for deriving structural information on protein complexes. However, modellers should prepare now for the arrival of large amounts of experimental data and fresh impetus to integrated structural biology from cross-linking/MS. The ease by which experimental data can be generated will ultimately have to be matched by the ease with which modelling tools can be operated, to allow integrated structural biology to spread throughout diverse fields in life sciences.
